# Subject-Specific Mapping of Excess Manganese Accumulation in the Brain of Welders Using Magnetic Resonance Imaging Relaxometry

**DOI:** 10.3390/toxics13030157

**Published:** 2025-02-25

**Authors:** Humberto Monsivais, Ulrike Dydak

**Affiliations:** 1 School of Health Sciences, Purdue University, West Lafayette, IN 47907, USA; hmonsiva@purdue.edu; 2 Department of Radiology and Imaging Sciences, Indiana University School of Medicine, Indianapolis, IN 46202, USA

**Keywords:** manganese, welding, MRI, R1 mapping, neurotoxicity

## Abstract

Chronic overexposure to manganese (Mn) can occur in occupational settings, such as welding, leading to increased Mn levels in the brain. Excess brain Mn accumulation may result in neurotoxicity, which is characterized by Parkinsonian-like symptoms including motor and cognitive dysfunctions. In this work, we demonstrate a novel methodology for personalized diagnosis and spatial characterization of abnormal Magnetic Resonance Imaging R1 (R1 = 1/T1) relaxation rates arising from excessive Mn accumulation in welders’ brains. Utilizing voxel-wise population-derived norms based on a frequency age-matched non-exposed group (n = 25), we demonstrate the ability to conduct subject-specific assessments and mapping of Mn exposure using MRI relaxometry. Our results show elevated R1 in multiple brain regions in individual welders, but also extreme between-subject variability in Mn accumulation, debasing the concept that high exposures correlate with uniformly high Mn deposition in the brain. Consequently, the presented personalized methodology serves as a counterpart to group-based comparison, which allows for understanding the level of individual exposure and the toxicokinetics of Mn accumulation. This work lays a foundation for improved occupational health assessments and preventive measures against neurotoxic metal exposure.

## 1. Introduction

Recent advancements in quantitative magnetic resonance imaging (qMRI) methods, which now have clinically practical acquisition durations, have made it possible to identify and analyze microstructural alterations in brain tissue in clinical research environments. In contrast to conventional MRI, where image contrasts are typically weighted towards one or more of the contrast mechanisms, qMRI quantifies these processes separately. Specifically, brain relaxometry measurements have shown their worth in various neurological disorders, as changes in the MR relaxation times T1 (longitudinal) and T2 (transverse) are associated with neuronal cell death, loss of axons, and demyelination processes [1,2,3]. This allows for tissue characterization and the potential to gain further insight into microstructural changes caused by diseases [4].

Manganese (Mn) is an essential trace element that serves as a coenzyme in processes like brain development, energy metabolism, bone growth, and cognitive function [5]. However, excessive environmental exposure, particularly in occupational settings, can cause neurotoxic effects, leading to a Parkinsonian disorder known as manganism. Symptoms include motor deficits, dystonia, rigidity, speech and mood disturbances [6,7,8], and cognitive impairment [9,10,11,12,13]. While past cases stemmed from acute, high-level exposure, modern exposure is typically low-level and chronic. Given this shift, manganism is less likely under current conditions, highlighting the need for more sensitive diagnostic methods to detect subtle Mn-induced neurotoxicity.

In the field of manganese (Mn) neurotoxicity, exposure to Mn and the resulting deposition of Mn in the brain has been shown to reduce the longitudinal relaxation time T1 in the brain, especially in the basal ganglia, leading to noticeable changes in the MRI signal in the globus pallidus [14,15,16]. A decrease in the T1 relaxation rate of tissue leads to an increase in signal intensity, referred to as “hyperintensity”, in T1-weighted (T1w) MR images. In workers exposed to Mn, an index known as the pallidal index (PI) has been used to assess exposure effects. This index calculates the ratio of T1w signal intensity in the globus pallidus (GP) to that in the frontal white matter (FWM) [17]. The PI has been found to correlate with various markers of Mn exposure, including blood Mn levels [18,19], cumulative exposure indices [20], and neurobehavioral performance [21]. However, the accuracy of the PI in indicating brain Mn levels is limited, as the accumulation of Mn in the white matter (WM) increases with higher levels of Mn exposure [6]. Furthermore, it only assumes Mn accumulation in the GP, yet deposition and clearance of Mn varies by brain region [22,23,24].

The paramagnetic properties of Mn can also be exploited via T1 mapping, a direct measurement of the T1 relaxation time, which may be more sensitive to brain Mn levels. Specifically, the longitudinal relaxation rate R1 (R1 = 1/T1) increases as Mn accumulation increases in the brain. Several research studies in welders exposed to Mn have employed T1 mapping, which, unlike the pallidal index, is less subjected to confounding factors [11,22,23,25,26]. However, most of these studies employed the widely adopted region of interest (ROI) analysis method. Although this method is effective for addressing specific questions, it offers only limited regional information and may overlook critical data outside the selected ROI. Additionally, all previous studies have examined group differences only. Group analyses help in identifying common patterns and trends across a population, have higher statistical power, and are effective in discovering biomarkers that are consistently present across different individuals. However, analysis at the individual level helps in identifying subtle or unique pathological features that might be overlooked in a group analysis (i.e., increased specificity), and allows monitoring of temporal changes in an individual, offering valuable information about the progression of a neurological condition, for example.

In the context of Mn exposure, an individual assessment approach can add a wealth of information since individual Mn exposure in the workplace varies greatly (e.g., open vs. closed spaces, years of exposure, manufacturing type, etc.). An individual based analysis can lead to a more holistic understanding of the toxicokinetics of excess Mn accumulation in the brain.

This work aims to create single-subject maps of elevated Mn in welders, by voxel-wise comparison to a normative atlas for R1 relaxation in the human brain from a cohort of factory controls. Within this context, the present study departs from a group-level analysis and applies a method for detecting and visualizing abnormal relaxation rates because of excess Mn accumulation in the *individual* brain of welders.

## 2. Materials and Methods

### 2.1. Data Acquisition

The present study utilized data previously collected by our research group at Purdue University [22,27,28]. Specifically for this analysis, subjects were limited to those who had a complete high-quality MRI and provided a complete medical and work history questionnaire, yielding a total of 36 welders and 25 controls (see Table 1 for more details). The research received approval from Purdue University’s Institutional Review Board (IRB-2023-1967), and all participants provided written informed consent before taking part in the study. Subjects with a history of Parkinson’s Disease or other motor disorders were excluded from participation. The study was completed in 2016; this analysis includes cross-sectional data collected from February 2013–May 2016.

### 2.2. Whole-Brain R1 Mapping

MRI imaging was conducted using a 3T GE Signa MRI scanner equipped with an 8-channel head coil. A 3D high-resolution T1-weighted imaging sequence, employing a fast-spoiled gradient-recalled echo (FSPGR) technique, was used for anatomical reference. The parameters were as follows: TR/TE at 6.54/2.8 ms, a flip angle of 12°, a 256 × 256 matrix, and 220 slices per volume, achieving a resolution of 0.9 × 0.9 × 1 mm^3^. T1 mapping was performed using a 3D spoiled gradient echo sequence with a variable flip angle (VFA) approach and two echoes (SPGR, TR/TE: 6.36/1.76 ms, flip angles α: 3°, 17°, resolution: 1 × 1 × 2 mm^3^). An inversion-recovery SPGR acquisition (IR-SPGR, TR/TE/IR = 6.36/1.76/250 ms, flip angle α: 3°, resolution: 1 × 1 × 2 mm^3^) was also included to correct for variations in flip angle [29]. R1 maps were generated using the two SPGR scans. The IR-SPGR scan served to adjust for variations in the flip angle. The fitting process utilized a specialized MATLAB (MathWorks, Natick, MA, USA) script, available at (https://github.com/mjt320/HIFI, accessed on 2 February 2022).

### 2.3. Data Processing for Normative R1 Atlas

**Figure 1:**
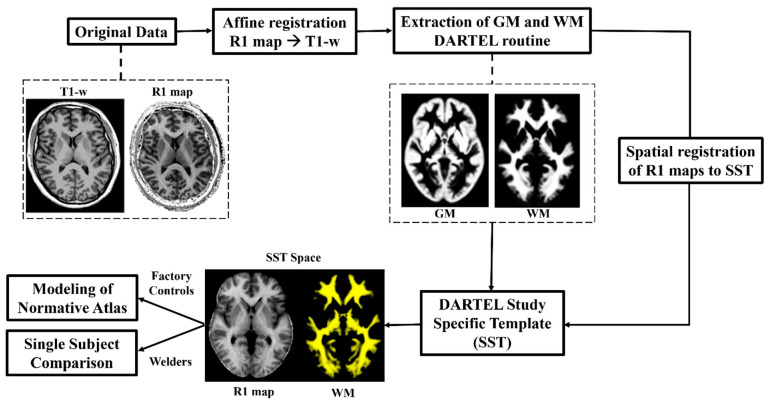
Pre-processing pipeline. First, the R1 map is rigidly registered to the T1-weighted structural image for each data set to correct for possible head motion between the two acquisitions. Then, gray matter (GM) and white matter (WM) probability maps are extracted via SPM segmentation to build the DARTEL study-specific template (SST). Finally, the skull-stripped R1 volumes are aligned with the SST via a nonlinear spatial registration; this transformation is then applied to the WM maps.

Image processing for establishing a normative R1 atlas followed similar steps as described in Monsivais et al. [24]. Briefly, R1 maps were coregistered to their corresponding T1w images using the normalized mutual algorithm in SPM12. Both the T1w and R1 volumes were automatically reoriented to the standard MNI space using the *AutoAlign* feature in the hMRI Toolbox [30]. Next, the T1w images were segmented into tissue classes following the DARTEL routine [31], which allows for the subsequent generation of a study-specific template (SST). The R1 maps were skull stripped using BET2 [32] in FSL [33]. A mean white matter (WM) mask was generated by averaging the normalized WM probability maps from the segmentation step and thresholding at values larger than 50% using the ImCal tool in SPM. Using a 50% threshold includes the GP (which is gray matter) but does not include the thalamus, putamen, and caudate nucleus. The skull-stripped R1 maps and mean WM mask were then nonlinear spatially registered to the DARTEL SST Processing steps before the computation of the normative atlas are summarized in Figure 1.

### 2.4. R1 Normative Atlas

A normative R1 atlas was established following the method of Piredda et al. [34]. In short, the relaxation rate variability within the healthy cohort (HC, 25 controls) was modeled using a linear regression model that accounts for the impact of age for each voxel position ($\vec{r}$):

(1) $$E\left\{R_{1}\left(\vec{r}\right)\right\}=\beta_{0,R_{1}}\left(\vec{r}\right)+\beta_{age,R_{1}}\left(\vec{r}\right)\cdot age+\beta_{age^{2},R_{1}}\left(\vec{r}\right)\cdot age^{2},$$

where *β* is the model intercept, and age in years (as a whole number) centered at the mean age of the HC group. A quadratic term was included to account for the inverse U-shape evolution of R1 with age in brain tissues [35,36,37]. A sex predictor can be added to the linear model; however, our study population is males only.

### 2.5. Single-Subject Comparison

Voxel-wise variations were calculated as z-scores against the established R1 normative atlas, resulting in a map that indicates the number of standard deviations each voxel’s R1 value deviates from the average. This method is outlined in the study by Piredda et al. [34]. Z-scores are computed for each voxel-position ($\vec{r}$) as follows:

$$zR_{1}\left(\vec{r}\right)=\frac{R_{1}\left(\vec{r}\right)-E\{R_{1}\left(\vec{r}\right)\}}{RMSE_{R_{1}}(\vec{r})}.$$

The root mean square error (RMSE) serves as an estimation of the standard deviation (SD) of the residual error across the linear model.

To aid in avoiding false positives, we performed a cluster correction for each z-score map in FSL using *fslcuster* with a threshold of z-scores > 6 and a minimum of 100 voxels.

## 3. Results

### 3.1. R1 Atlas

**Figure 2:**
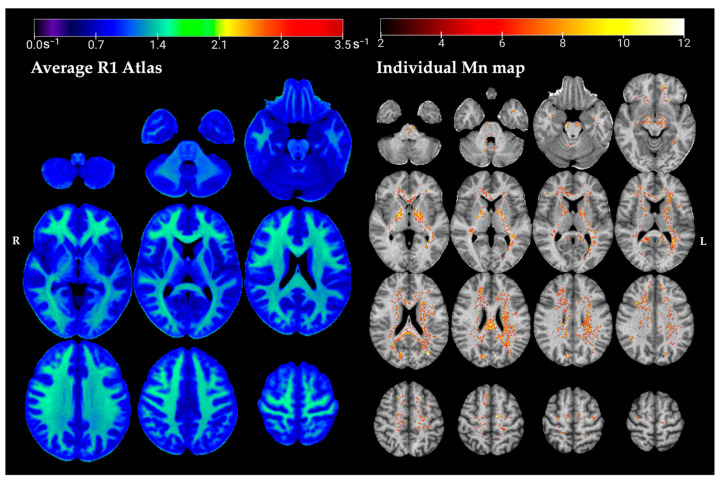
Axial slices of the normative R1 atlas (**left** panel). These spatial maps of the intercept coefficients correspond to the average R1 at the mean age of the HC (39 years). The z-scores map is overlayed onto the R1 map of an individual welder (**right** panel), representing areas of excess Mn accumulation in this individual’s brain.

The axial slices of the normative R1 map and an example individual Mn map are presented in Figure 2. The average R1 map corresponds to the intercept coefficient *β* of $E\left\{R_{1}\left(\vec{r}\right)\right\}=\beta_{0,R_{1}}\left(\vec{r}\right)+\beta_{age,R_{1}}\left(\vec{r}\right)\cdot age+\beta_{age^{2},R_{1}}\left(\vec{r}\right)\cdot age^{2}$ for the healthy control group (HC) at their mean age of 39 years. At the mean age of 39, the expected R1 in a voxel of the white matter (WM) in the frontal lobe was E{R_1_} ± RMSE = 1.07 ± 0.15 s^−1^, and 1.10 ± 0.09 s^−1^ in the GP.

### 3.2. Case Reports

**Figure 3:**
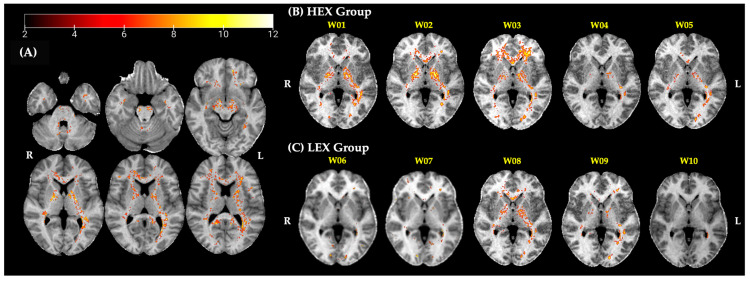
(**A**) Axial slices showcasing an individual welder’s R1 map with the corresponding z-score map (representative of excess Mn accumulation) overlaid on it. Selected examples of z-score maps on individual (**B**) HEX and (**C**) LEX welders’ brains reveal widespread increased z-score values throughout much of the cerebral white matter and deep brain structures. Note that cortical gray matter was not included in the normative maps.

Z-scores maps of ten selected cases (W01–W10) of five high exposure (HEX) and five low exposure (LEX) welders are presented in Figure 3. The HEX and LEX assignment is based on the past 3 months of individual exposure levels as described in Ma et al. [27]. Briefly, a model was created to calculate the cumulative exposure index (CEI) for each participant, serving as an estimate of the individual’s cumulative exposure to respirable airborne manganese (Mn) with a diameter of less than 4 μm over various time periods. This estimate was based on personal air samples, as explained by Ward et al. [28]. Welders who had exposure levels exceeding 0.04 (mg/m^3^)·year in the past three months (CEI_3M_) were classified into the HEX subgroup, indicating they had an average annual exposure to airborne manganese (Mn) levels of 0.16 mg/m^3^. Exposure characteristics for the ten selected welders are presented in Table 2.

Interestingly, not all welders in the HEX group showed widespread excess Mn accumulation, and some welders in the LEX group showed similar patterns of excess Mn accumulation as in the HEX group.

## 4. Discussion

The results from this study support the feasibility of a personalized (i.e., subject-specific) detection and characterization of abnormal R1 relaxation rates due to excess Mn accumulation in the brain of welders. Voxel-wise population-derived norms were established by modeling R1 distribution values (proxy for Mn concentrations) within a frequency age-matched non-exposed group following the proof-of-concept method in Piredda et al. [34]. When considering exposure to Mn, adopting an approach that assesses individuals can significantly enrich our understanding due to the considerable variation in exposure levels within the workplace (such as differences in open versus closed environments, duration of exposure, types of manufacturing, and respirator use). By focusing on individual assessments, we can achieve a more comprehensive grasp of how excess Mn accumulates in the brain and its toxicokinetics. Notably, occupational exposure is not the sole pathway for excess Mn in the brain; conditions such as liver dysfunction (e.g., cirrhosis) can also lead to abnormal Mn accumulation in areas like the GP and basal ganglia [38,39]. The liver’s role in regulating Mn levels through endogenous gut losses is disrupted when liver transporters (e.g., DMT1, Tf/TfR) and exporters (e.g., SLC30A10, ferroportin) malfunction [40], while factors like iron status and diet further influence Mn uptake [41]. Additionally, recent findings suggest that alcohol exposure may alter Mn transport and neurotoxicity in the brain [42]. These various factors underscore the importance of considering a wide range of individual and environmental factors when assessing Mn accumulation.

This method departs from conventional group comparisons (most used for increased sensitivity and statistical power) and provides a personalized assessment about relaxation alterations due to Mn exposure on an individual basis. Similarly to the results by Piredda et al. [34], the low false-positive rate (FPR) in WM assessed in the non-exposed group with a k = 10-fold cross-validation (mean FPR = 7.01%) attests to the robustness of the method (see Supplementary Materials Figure S1). The restriction of the z-score calculation to WM and GP is based on the RMSE results which showed that the highest error occurred in the cortical GM structures due to much more pronounced anatomic variation leading to imperfect spatial normalization. In other words, when normalizing the R1 maps to the SST, this imperfect alignment of cortical areas between subjects leads to a blended distribution of WM and GM within these voxels, making it difficult to interpret the findings in analyses that focus on individual subjects [43]. Future efforts should concentrate on enhancing the spatial alignment of the cortex. Recent advances in image processing methods have led to the development of novel methods based on deep learning approaches [44,45,46] that could enhance the spatial alignment of the cortex; however, these approaches are complex and require high computational performance. For this study, we used publicly available software (SPM12 v12.6) with the goal of improving accessibility and reproducibility of the method and assessing its use in studying brain Mn deposition due to welding.

Overall, our results suggest that even workers who do the same type of welding may have very differing Mn accumulation maps and that a high recent exposure (CEI_3M_) or high cumulative exposure (CEI_Life_) does not always translate to increased excess accumulation of Mn in the brain (see Figure 3 and Table 2). For example, subjects W02 and W03, although both having a CEI_3M_ of 0.05 mg/m^3^·yr and belonging to the HEX group, do not show the same pattern of Mn accumulation. It even appears that W03 with only 3 welding years and a much lower CEI_Life_ than W02 has more widespread Mn accumulation in the brain. In contrast, W08 shows similar patterns of accumulation to W01 and W02 in the HEX group. Although W08 is in the LEX group due to their CEI_3M_, this subject has a relatively high cumulative exposure in their working lifetime (CEI_Life_). These observations indicate that recent exposure, welding years, or age alone do not fully account for the patterns of Mn accumulation observed in these welders, with potential explanations discussed below. The overall structure of our subject-specific results seems to agree with the spatial deposition seen in our group analysis, where we demonstrated that excess accumulation of Mn in the brain spreads beyond the basal ganglia through white matter tracts, reaching brain regions linked to motor and cognitive abilities [24]. As expected, we observe a high accumulation of Mn in the GP in several welders and some show a similar distribution pattern as seen in our group-based analysis (e.g., W01–03, and W08) [24]. This aligns with that reported in the literature about Mn transport into the brain. Mn can penetrate the blood–brain barrier (BBB) through transferrin receptor-mediated endocytosis, a process prominently active in the basal ganglia [47]. This mechanism increases the vulnerability of these areas to Mn buildup. Additionally, the basal ganglia’s elevated energy requirements and metabolic rate can affect the transportation and accumulation of metals like iron, which share similar chemical properties and use the same transport mechanisms [48].

In this cohort, Mn air levels must surpass 0.1 mg/m^3^ for the brain maps to show elevated Mn levels, as W06, W07, and W10 do not show any accumulation. Yet the amount and pattern of deposition seem to further depend on other factors like the time of welding, respirator use, and overall cumulative exposure. The most drastic difference seen in our HEX group is W03 whose Mn maps show a significant amount of Mn accumulation in the frontal white matter (FWM). W03 has been welding for only 3 years and has a high recent exposure (CEI_3M_ = 0.05 mg/m^3^·yr) similar to W02 and W04, and the lowest CEI_Life_ amongst the HEX welders. However, this welder demonstrates the most widespread Mn accumulation, reaching all the way to the frontal white matter. Findings by Edmondson et al. suggest that uptake and clearance vary according to the region of the brain and are also dependent on CEI_Life_ with lower CEI_Life_ leading to faster uptake [22], which may explain why a welder with high recent exposure and low cumulative exposure shows higher Mn deposition in their brain. On the other hand, W10 has a similar CEI_Life_, with about half the CEI_3M_ of W03, yet shows no Mn accumulation at all, again demonstrating that no known sole factor about these welders explains all the patterns. Another consideration could be alcohol consumption: W03 self-reported 5–10 alcoholic drinks per week compared to 0–3 drinks reported by the rest of the cohort. While self-reported alcohol use is often an underestimation [49], it might point to the higher alcohol consumption of W03 compared to the others. A recent study in mice by Han et al. showed that alcohol consumption in mice leads to an increased hypoxic response and a reduction in hepcidin expression, which in turn provides the molecular basis for the enhanced activity of iron transporters and increased Mn uptake associated with alcohol intake [42]. Ellingsen et al. demonstrated that welders who consumed alcohol performed worse in neurobehavioral tests compared to referents, indicating a significant interaction between alcohol consumption and Mn exposure on brain function [50].

However, a limitation common to many studies on occupational Mn exposure, including this one, is the absence of detailed data on individual exposure levels. While we are one of few studies measuring personal air samples, and our exposure model is robust, it relies on a single time point of exposure measurement that is extrapolated across other time windows based on work histories and measurements from other working departments. Ideally, to better assess variation in daily exposure, workers should be monitored over multiple workdays, and more detailed data on respiratory protection use should be collected alongside assessments of personal airborne Mn exposure.

The lack of a clear explanation or threshold in terms of any of our exposure measures (Mn air levels, cumulative exposure, recent exposure, years of exposure) demonstrates clearly how the amount and pattern of Mn accumulation in the human brain differ between individuals, supporting the need for individual measures and visualization of Mn accumulation, as presented in this paper.

The strength of this novel method of visualizing Mn deposition includes the potential for monitoring spatial-temporal changes in workers who have just started their welding career or those who will be exposed to airborne Mn particles in general (such as miners). Furthermore, the same approach can be applied to monitor the safety of repeated MRI scans using gadolinium (Gd)-based contrast agents (another toxic metal that affects R1 in MRI) since growing concerns have arisen from research indicating the accumulation of Gd in different brain areas of patients who have had multiple Gd-based MRI scans [51,52]. Lastly, R2 or R2* atlases can be established to study the accumulation dynamics of iron, a significant constituent of welding fumes, using this method.

The main limitation of our current established normative R1 atlas is the number of control subjects in the HC. Additionally, our normative R1 atlas is based on a male-only population; however, this only limits the broader generatability of the results since our study data in this cohort consisted of male welders only. Therefore, current efforts are focused on broadening the scope and diversity of the HC data set by incorporating a larger sample size that encompasses both sexes to develop the R1 atlas, which will allow us to also study female welders and investigate sex differences. Previous research has shown that both sex and normal aging have an impact on T1 [34,35,36]. Age differences in our current cohort likely do not account for the observed R1 patterns, as subjects’ ages span above and below the mean of 39 years, suggesting age alone does not drive our findings. It is worth noting that a regression model to assess if R1 in any voxels was associated with age showed that only voxels within the caudate nucleus were positively associated with age; however, this did not hold after correcting for multiple comparisons (see Supplementary Materials Figure S2). Lastly, other subcortical areas of the basal ganglia such as the thalamus, putamen, and caudate nucleus were not included in the mask for z-score calculations since these are gray matter structures that suffer from imperfect alignment of cortical areas as discussed above. Thus, the findings from this study should not be interpreted as if no Mn accumulation occurs in these areas. Additionally, this study did not shed light on cortical gray matter areas or the cerebellum, which will benefit from the development of more sophisticated methods.

## 5. Conclusions

In conclusion, this study demonstrates the feasibility and potential significance of individualized evaluations of increased R1 relaxation rates as indicative of excessive manganese accumulation in the brain of welders. With the aid of a frequency age-matched non-exposed group derived from the same population, we established voxel-wise population-based norms to implement a framework for non-averaged individual evaluation. This novel strategy illustrates the wide-ranging disparity in manganese accumulation levels and patterns. Our findings show that high recent or cumulative Mn exposure does not consistently result in greater brain accumulation, revealing the complexity of Mn toxicokinetics. This emphasizes the role of other confounding factors that influence individual Mn exposure and brain deposition, demonstrating the need for personalized assessment methods. Future research utilizing these individualized techniques will aim to examine these confounding factors in greater detail as well as to study uptake and washout dynamics in different brain regions.

## Figures and Tables

**Table 1:** Characteristics of participants included in the analysis.

Characteristics of Participants	Welders (n = 36)	Controls (n = 25)
Age (y) [mean ± SD], range	39.9 ± 10.7, (21, 57)	38.9 ± 11.1, (20, 61)
Years of Education (y) [mean ± SD], range	12.6 ± 1.3, (8, 12)	12.9 ± 1.2, (12, 16)
Welding years (y) [mean ± SD], range	12.5 ± 8.8, (1.7, 36)	0 ± 0, -
**Exposure (indices are given in units of mg/m^3^·yr) [mean ± SD], range**		
Mean Airborne Mn Exposure (mg/m^3^)	0.146 ± 0.109, (0.051, 0.477)	0.005 ± 0.008, (0, 0.027)
Mn cumulative exposure index (Mn-CEI_3M_)	0.038 ± 0.035, (0.006, 0.179)	0.0005 ± 0.0003, (0, 0.006)
Mn cumulative exposure index (Mn-CEI_7-12M_)	0.074 ± 0.080, (0.009, 0.404)	0.001 ± 0.001, (0, 0.013)
Mn cumulative exposure index (Mn-CEI_Life_)	1.444 ± 1.292, (0.026, 4.807)	0.059 ± 0.081, (0.003, 0.403)

**Table 2:** Age and occupational characteristics from the ten selected welders (W01–W10) shown in Figure 3. Exposure metrics include past three months exposure (CEI_3M_) and cumulative exposure over their working lifetime (back to age 18, CEI_Life_).

**HEX Group**					
	W01	W02	W03	W04	W05
Age [yrs]	48	50	34	56	29
Race	White	White	Hispanic	White	White
Welding time [yrs]	11	24	3	36	6
Welding type ^†^	MIG-MS	MIG-MS	MIG-MS	MIG-MS	MIG-MS
Air Mn (mg/m^3^)	0.16	0.12	0.12	0.48	0.48
CEI_3M_ [mg/m^3^·yr]	0.12	0.05	0.05	0.05	0.06
CEI_Life_ [mg/m^3^·yr]	1.49	2.44	0.36	4.8	0.93
**LEX Group**					
	W06	W07	W08	W09	W10
Age [yrs]	37	37	46	31	29
Race	Hispanic	African Am.	African Am.	White	African Am.
Welding time [yrs]	11	14	21	7	4
Welding type ^†^	MIG-MS	MIG-MS	MIG-HSS	MIG-HSS	MIG-MS
Air Mn (mg/m^3^)	0.08	0.08	0.15	0.20	0.07
CEI_3M_ [mg/m^3^·yr]	0.04	0.01	0.02	0.02	0.03
CEI_Life_ [mg/m^3^·yr]	0.56	0.68	1.37	0.51	0.31

^†^ Metal Inert Gas Mild Steel (MIG-MS) and high strength steel (MIG-HSS).

## Data Availability

The data related to this article is not publicly available, but the methodology makes use of a publicly available toolbox (SPM12, www.fil.ion.ucl.ac.uk/spm, accessed on 2 February 2022). The standardized procedures (tissue segmentation, DARTEL, voxel-based quantification) within this toolbox were followed. The Matlab script for generating the relaxometry maps is publicly available on GitHub (https://github.com/mjt320/HIFI, accessed on 2 February 2022). The only modifications to this script were the parameters for our MRI acquisition (e.g., echo times, flip angles, inversion recovering time).

## Supplementary Materials

The following supporting information can be downloaded at: https://www.mdpi.com/article/10.3390/toxics13030157/s1, Figure S1: False-positive rate (FPR) map in WM assessed in the non-exposed group with a k = 10-fold cross-validation (mean FPR = 7.01%); Figure S2: Linear regression model to assess if any voxels were associated with age. Voxels within the caudate nucleus and sparse areas in white matter were positively associated with age; however, this did not hold after correcting for multiple comparisons.

## References

[1] Dean D.C., Sojkova J., Hurley S., Kecskemeti S., Okonkwo O., Bendlin B.B., Theisen F., Johnson S.C., Alexander A.L., Gallagher C.L. (2016). Alterations of Myelin Content in Parkinson’s Disease: A Cross-Sectional Neuroimaging Study. PLoS ONE.

[2] Baudrexel S., Nürnberger L., Rüb U., Seifried C., Klein J.C., Deller T., Steinmetz H., Deichmann R., Hilker R. (2010). Quantitative Mapping of T1 and T2* Discloses Nigral and Brainstem Pathology in Early Parkinson’s Disease. NeuroImage.

[3] Bonnier G., Fischi-Gomez E., Roche A., Hilbert T., Kober T., Krueger G., Granziera C. (2018). Personalized Pathology Maps to Quantify Diffuse and Focal Brain Damage. NeuroImage Clin..

[4] Weiskopf N., Mohammadi S., Lutti A., Callaghan M.F. (2015). Advances in MRI-Based Computational Neuroanatomy: From Morphometry to in-Vivo Histology. Curr. Opin. Neurol..

[5] Aschner M., Erikson K. (2017). Manganese. Adv. Nutr..

[6] Guilarte T.R., Gonzales K.K. (2015). Manganese-Induced Parkinsonism Is Not Idiopathic Parkinson’s Disease: Environmental and Genetic Evidence. Toxicol. Sci..

[7] Racette B.A., Aschner M., Guilarte T.R., Dydak U., Criswell S.R., Zheng W. (2012). Pathophysiology of Manganese-Associated Neurotoxicity. NeuroToxicology.

[8] Tsuboi Y., Uchikado H., Dickson D.W. (2007). Neuropathology of Parkinson’s Disease Dementia and Dementia with Lewy Bodies with Reference to Striatal Pathology. Park. Relat. Disord..

[9] Bowler R.M., Nakagawa S., Drezgic M., Roels H.A., Park R.M., Diamond E., Mergler D., Bouchard M., Bowler R.P., Koller W. (2007). Sequelae of Fume Exposure in Confined Space Welding: A Neurological and Neuropsychological Case Series. NeuroToxicology.

[10] Bowler R.M., Lezak M.D. (2015). Neuropsychologic Evaluation and Exposure to Neurotoxicants. Handbook of Clinical Neurology.

[11] Bowler R.M., Yeh C.L., Adams S.W., Ward E.J., Ma R.E., Dharmadhikari S., Snyder S.A., Zauber S.E., Wright C.W., Dydak U. (2018). Association of MRI T1 Relaxation Time with Neuropsychological Test Performance in Manganese-Exposed Welders. NeuroToxicology.

[12] Zoni S., Albini E., Lucchini R. (2007). Neuropsychological Testing for the Assessment of Manganese Neurotoxicity: A Review and a Proposal. Am. J. Ind. Med..

[13] Martin K.V., Edmondson D., Cecil K.M., Bezi C., Vance M.L., McBride D., Haynes E.N. (2020). Manganese Exposure and Neurologic Outcomes in Adult Populations. Neurol. Clin..

[14] Kim Y., Kim K.S., Yang J.S., Park I.J., Kim E., Jin Y., Kwon K.R., Chang K.H., Kim J.W., Park S.H. (1999). Increase in Signal Intensities on T1-Weighted Magnetic Resonance Images in Asymptomatic Manganese-Exposed Workers. Neurotoxicology.

[15] Criswell S.R., Perlmutter J.S., Huang J.L., Golchin N., Flores H.P., Hobson A., Aschner M., Erikson K.M., Checkoway H., Racette B.A. (2012). Basal Ganglia Intensity Indices and Diffusion Weighted Imaging in Manganese-Exposed Welders. Occup. Environ. Med..

[16] Dydak U., Criswell S.R. (2014). Chapter 19—Imaging Modalities for Manganese Toxicity. Manganese in Health and Disease.

[17] Krieger D., Krieger G.S., Theilmann L., Krieger D. (1995). Manganese and Chronic Hepatic Encephalopathy Departments of Neurology. The Lancet.

[18] Chang Y., Woo S.T., Kim Y., Lee J.J., Song H.J., Lee H.J., Kim S.H., Lee H., Kwon Y.J., Ahn J.H. (2010). Pallidal Index Measured with Three-Dimensional T1-Weighted Gradient Echo Sequence Is a Good Predictor of Manganese Exposure in Welders. J. Magn. Reson. Imaging.

[19] Jiang Y., Zheng W., Long L., Zhao W., Li X., Mo X., Lu J., Fu X., Li W., Liu S. (2007). Brain Magnetic Resonance Imaging and Manganese Concentrations in Red Blood Cells of Smelting Workers: Search for Biomarkers of Manganese Exposure. NeuroToxicology.

[20] Dietz M.C., Ihrig A., Wrazidlo W., Bader M., Jansen O., Triebig G. (2001). Results of Magnetic Resonance Imaging in Long-Term Manganese Dioxide-Exposed Workers. Environ. Res..

[21] Shin Y.C., Kim E., Cheong H.K., Cho S., Sakong J., Kim K.S., Yang J.S., Jin Y.W., Kang S.K., Kim Y. (2007). High Signal Intensity on Magnetic Resonance Imaging as a Predictor of Neurobehavioral Performance of Workers Exposed to Manganese. NeuroToxicology.

[22] Edmondson D.A., Ma R.E., Yeh C.-L., Ward E., Snyder S., Azizi E., Zauber S.E., Wells E.M., Dydak U. (2019). Reversibility of Neuroimaging Markers Influenced by Lifetime Occupational Manganese Exposure. Toxicol. Sci..

[23] Edmondson D.A., Yeh C.L., Hélie S., Dydak U. (2020). Whole-Brain R1 Predicts Manganese Exposure and Biological Effects in Welders. Arch. Toxicol..

[24] Monsivais H., Yeh C.-L., Edmondson A., Harold R., Snyder S., Wells E.M., Schmidt-Wilcke T., Foti D., Zauber S.E., Dydak U. (2024). Whole-Brain Mapping of Increased Manganese Levels in Welders and Its Association with Exposure and Motor Function. NeuroImage.

[25] Lee E.Y., Flynn M.R., Du G., Lewis M.M., Fry R., Herring A.H., Van Buren E., Van Buren S., Smeester L., Kong L. (2015). T1 Relaxation Rate (R1) Indicates Nonlinear Mn Accumulation in Brain Tissue of Welders with Low-Level Exposure. Toxicol. Sci..

[26] Choi D.S., Kim E.A., Cheong H.K., Khang H.S., Ryoo J.W., Cho J.M., Sakong J., Park I. (2007). Evaluation of MR Signal Index for the Assessment of Occupational Manganese Exposure of Welders by Measurement of Local Proton T1 Relaxation Time. NeuroToxicology.

[27] Ma R.E., Ward E.J., Yeh C.L., Snyder S., Long Z., Gokalp Yavuz F., Zauber S.E., Dydak U. (2018). Thalamic GABA Levels and Occupational Manganese Neurotoxicity: Association with Exposure Levels and Brain MRI. NeuroToxicology.

[28] Ward E.J., Edmondson D.A., Nour M.M., Snyder S., Rosenthal F.S., Dydak U. (2018). Toenail Manganese: A Sensitive and Specific Biomarker of Exposure to Manganese in Career Welders. Ann. Work Expo. Health.

[29] Deoni S.C.L., Rutt B.K., Peters T.M. (2003). Rapid Combined T1 and T2 Mapping Using Gradient Recalled Acquisition in the Steady State. Magn. Reson. Med..

[30] Tabelow K., Balteau E., Ashburner J., Callaghan M.F., Draganski B., Helms G., Kherif F., Leutritz T., Lutti A., Phillips C. (2019). hMRI—A Toolbox for Quantitative MRI in Neuroscience and Clinical Research. NeuroImage.

[31] Ashburner J. (2007). A Fast Diffeomorphic Image Registration Algorithm. NeuroImage.

[32] Jenkinson M., Pechaud M., Smith S. (2005). BET2—MR-Based Estimation of Brain, Skull and Scalp Surfaces. Elev. Annu. Meet. Organ. Hum. Brain Mapp..

[33] Jenkinson M., Beckmann C.F., Behrens T.E.J., Woolrich M.W., Smith S.M. (2012). FSL. NeuroImage.

[34] Piredda G.F., Hilbert T., Granziera C., Bonnier G., Meuli R., Molinari F., Thiran J.P., Kober T. (2020). Quantitative Brain Relaxation Atlases for Personalized Detection and Characterization of Brain Pathology. Magn. Reson. Med..

[35] Badve C., Yu A., Rogers M., Ma D., Liu Y., Schluchter M., Sunshine J., Griswold M., Gulani V. (2015). Simultaneous T1 and T2 Brain Relaxometry in Asymptomatic Volunteers Using Magnetic Resonance Fingerprinting. Tomography.

[36] Draganski B., Ashburner J., Hutton C., Kherif F., Frackowiak R.S.J., Helms G., Weiskopf N. (2011). Regional Specificity of MRI Contrast Parameter Changes in Normal Ageing Revealed by Voxel-Based Quantification (VBQ). Neuroimage.

[37] Slater D.A., Melie-Garcia L., Preisig M., Kherif F., Lutti A., Draganski B. (2019). Evolution of White Matter Tract Microstructure across the Life Span. Hum. Brain Mapp..

[38] Abenavoli L., Fabiano G., Procopio A.C., Aquila I., Pellicano R., Barone S., Morelli M. (2022). Hepatic Encephalopathy by Manganese Deposition: A Case Report and a Review of Literature. Rev. Recent Clin. Trials.

[39] Kano Y., Morishima R. (2021). Pallidal Manganese Concentration in Hepatic Encephalopathy. Clin. Gastroenterol. Hepatol..

[40] Chen P., Bornhorst J.B., Aschner M. (2018). Manganese Metabolism in Humans. Front. Biosci. Landmark.

[41] Ye Q., Park J.E., Gugnani K., Betharia S., Pino-Figueroa A., Kim J. (2017). Influence of Iron Metabolism on Manganese Transport and Toxicity. Metallomics.

[42] Han M., Böhlke M., Maher T., Kim J. (2021). Alcohol Exposure Increases Manganese Accumulation in the Brain and Exacerbates Manganese-Induced Neurotoxicity in Mice. Arch. Toxicol..

[43] Scarpazza C., Sartori G., De Simone M.S., Mechelli A. (2013). When the Single Matters More than the Group: Very High False Positive Rates in Single Case Voxel Based Morphometry. NeuroImage.

[44] Zhang S., Liu P.X., Zheng M., Shi W. (2020). A Diffeomorphic Unsupervised Method for Deformable Soft Tissue Image Registration. Comput. Biol. Med..

[45] Balakrishnan G., Zhao A., Sabuncu M.R., Guttag J., Dalca A.V. (2019). VoxelMorph: A Learning Framework for Deformable Medical Image Registration. IEEE Trans. Med. Imag..

[46] Shao S., Pei Z., Chen W., Zhu W., Wu X., Zhang B. (2022). A Multi-Scale Unsupervised Learning for Deformable Image Registration. Int. J. Comput. Assist. Radiol. Surg..

[47] Rabin O., Hegedus L., Bourre J.-M., Smith Q.R. (1993). Rapid Brain Uptake of Manganese (II) Across the Blood-Brain Barrier. J. Neurochem..

[48] Prohaska J.R. (1987). Functions of Trace Elements in Brain Metabolism. Physiol. Rev..

[49] Stockwell T., Zhao J., Greenfield T., Li J., Livingston M., Meng Y. (2016). Estimating Under- and over-Reporting of Drinking in National Surveys of Alcohol Consumption: Identification of Consistent Biases across Four English-Speaking Countries. Addict. Abingdon Engl..

[50] Ellingsen D.G., Kusraeva Z., Bast-Pettersen R., Zibarev E., Chashchin M., Thomassen Y., Chashchin V. (2014). The Interaction between Manganese Exposure and Alcohol on Neurobehavioral Outcomes in Welders. Neurotoxicol. Teratol..

[51] McDonald R.J., McDonald J.S., Kallmes D.F., Jentoft M.E., Murray D.L., Thielen K.R., Williamson E.E., Eckel L.J. (2015). Intracranial Gadolinium Deposition after Contrast-Enhanced MR Imaging. Radiology.

[52] Kanda T., Fukusato T., Matsuda M., Toyoda K., Oba H., Kotoku J., Haruyama T., Kitajima K., Furui S. (2015). Gadolinium-Based Contrast Agent Accumulates in the Brain Even in Subjects without Severe Renal Dysfunction: Evaluation of Autopsy Brain Specimens with Inductively Coupled Plasma Mass Spectroscopy. Radiology.

