# Supplementary material for: Subject-Specific Mapping of Excess Manganese Accumulation in the Brain of Welders Using Magnetic Resonance Imaging Relaxometry
Source: Toxics. 2025 Feb 25;13(3):157. doi: 10.3390/toxics13030157 (PMC11945464; doi:10.3390/toxics13030157)
Supplement: Supplementary file 1 [file toxics-13-00157-s001.zip › Individual-Mn-maps_manuscript_SuppMaterials.pdf]

toxics

MDPI

Supplementary Materials

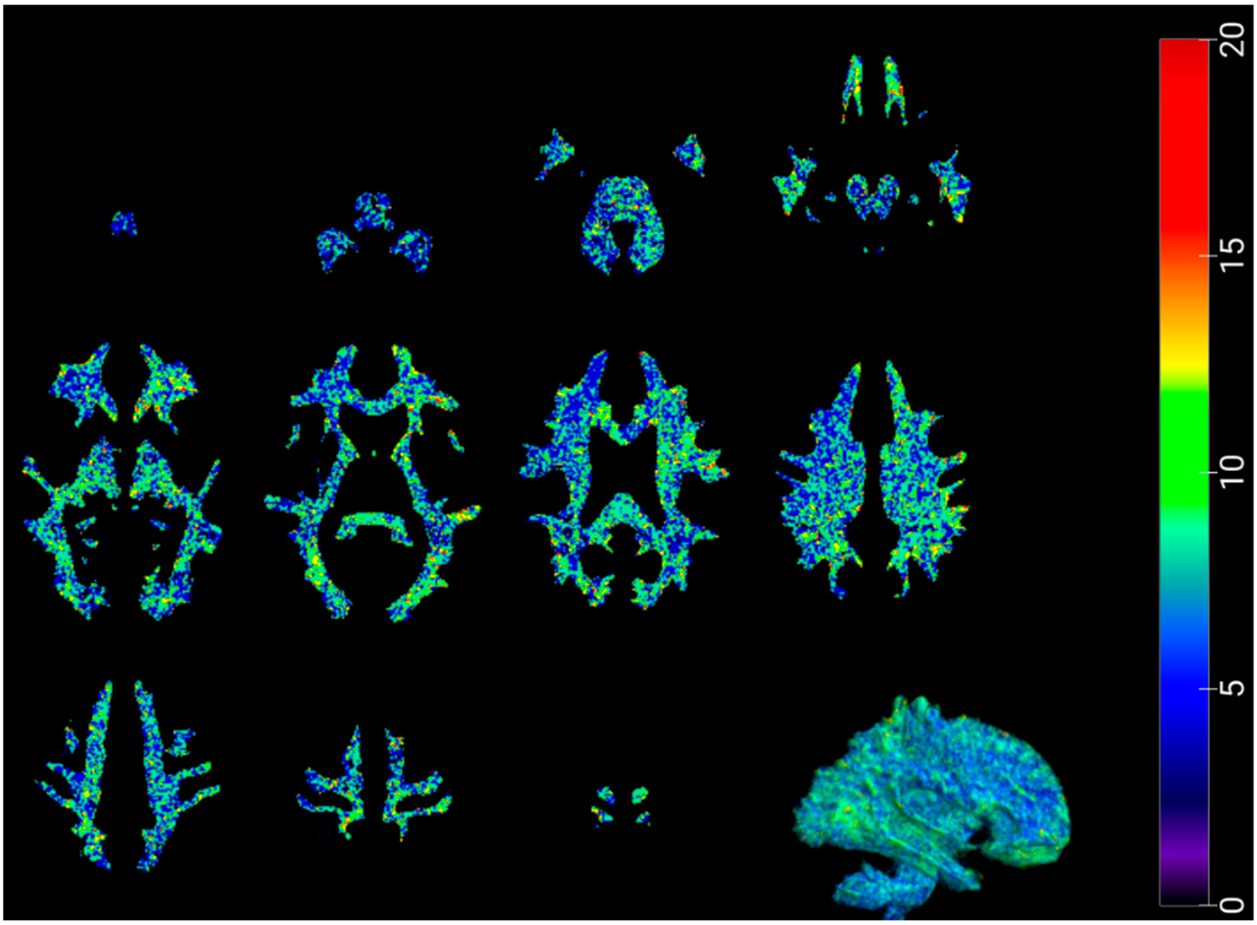

Figure S1: False-positive rate (FPR) map in WM assessed in the non-exposed group with a k-10 fold cross validation (mean FPR = 7.01%).

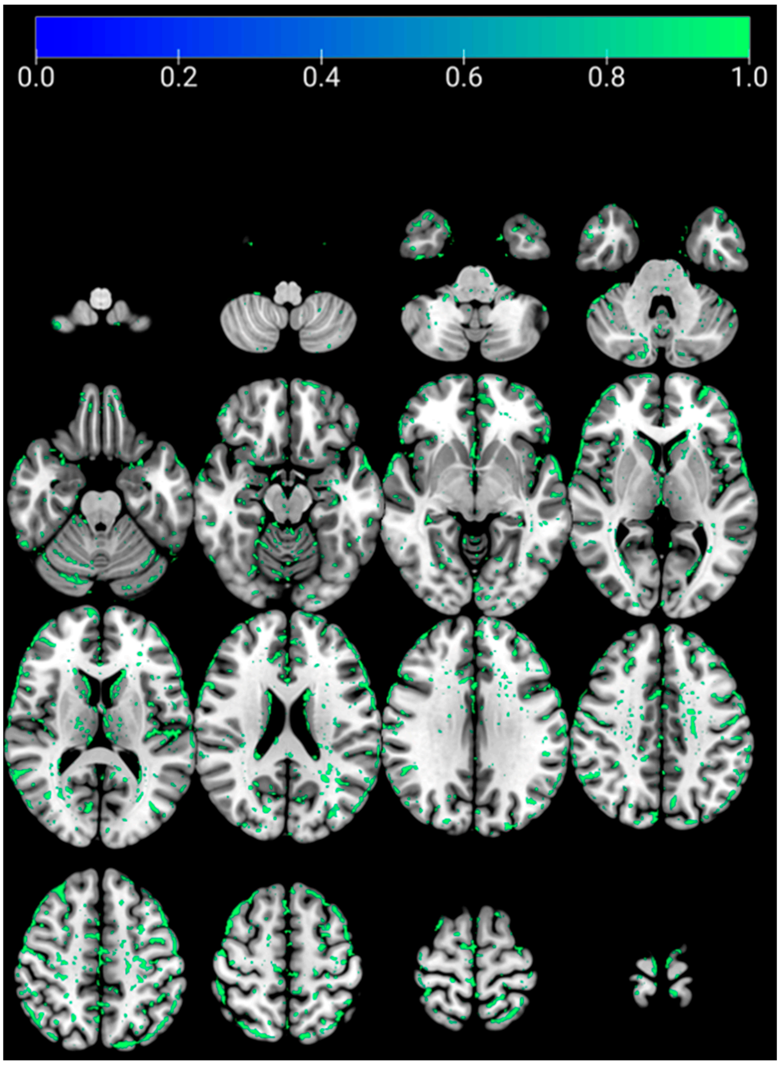

Figure S2: Linear regression model to assess if R1 values in any voxels were associated with age. Voxels within the caudate nucleus and sparse areas in white matter were positively associated with age, however, this did not hold after correcting for multiple comparisons.
